# Cannabidiol (CBD) for the treatment of subcutaneous treprostinil (Remodulin^®^) site pain: a case report

**DOI:** 10.3389/fmed.2023.1188083

**Published:** 2023-06-13

**Authors:** Jacqueline Brewer, Amy Kimber

**Affiliations:** ^1^Beaumont Health, Troy, MI, United States; ^2^United Therapeutics Corporation, Research Triangle Park, NC, United States

**Keywords:** pulmonary arterial hypertension, treprostinil, subcutaneous infusion, cannabinoids, pain, pain management (MeSH), injection site reaction

## Abstract

**Background:**

Pulmonary arterial hypertension occurs as a result of vascular remodeling and dysregulation of endothelial cells that narrows small pulmonary arteries and raises precapillary pressures. Pulmonary arterial hypertension is a rare and progressive disease characterized by dyspnea, chest pain, and syncope. Parenteral treprostinil is indicated for the treatment of pulmonary arterial hypertension to diminish symptoms associated with exercise. Up to 92% of patients treated with treprostinil via subcutaneous delivery experienced infusion site pain and approximately 23% discontinued treatment due to site pain. Cannabidiol salve may have analgesic and anti-inflammatory properties and could be an additional option for patients with infusion site pain.

**Case report:**

Two patients with pulmonary arterial hypertension were treated with cannabidiol salve. Both patients reported a reduction in infusion site pain without the need for narcotics.

**Conclusion:**

These two cases suggest that cannabidiol salve may help to minimize redness and alleviate pain at the infusion site. Additional studies are required to test the effectiveness of cannabidiol in a larger group of patients with infusion site pain.

## Introduction

Pulmonary arterial hypertension (PAH) occurs as a result of vascular remodeling and dysregulation of endothelial cells that narrows small pulmonary arteries and raises precapillary pressures (1). PAH is a rare and progressive disease with no known cure, which is characterized by dyspnea, chest pain, and syncope that can lead to right ventricular dysfunction, eventually progressing to right heart failure and often death (1, 2). Hemodynamically, PAH is defined as a mean pulmonary arterial pressure (mPAP) of >20 mmHg, a pulmonary vascular resistance (PVR) of ≥2 wood units, and a pulmonary capillary wedge pressure of ≤15 mmHg (3). An imbalance of endothelin-1, nitric oxide, and prostacyclin pathways contributes to disease development and are the main pathways targeted by current FDA-approved medications for PAH (1, 4).

Subcutaneous (SC) treprostinil (Remodulin^®^), approved in 2002, is a prostacyclin analog indicated for the treatment of PAH (Group 1 pulmonary hypertension) to diminish symptoms associated with exercise (5). Treprostinil causes direct vasodilation of pulmonary and systemic arterial vascular beds and inhibits platelet aggregation (6–8). Continuous treprostinil infusions can be delivered by a subcutaneous or intravenous (IV) route. IV infusions require a central venous catheter for administration or a peripherally inserted central catheter (PICC) for short-term infusions (9). Central venous catheters are associated with the risk of serious complications, such as bloodstream infections and sepsis, making SC delivery the preferred route (10). The most common adverse side effect of SC administration is infusion site pain and reaction, which, in the pivotal, randomized, placebo-controlled trial, led to the discontinuation of therapy in 8% of patients (10).

Infusion site pain and reaction from SC treprostinil may be characterized by mild-to-moderate-to-severe inflammation, tenderness, surrounding erythema, mild bleeding, and nodularity or induration (11). The mechanism is unknown; however, potential causes may include inflammation, vasodilation, and pain stimulation (11). The SC site pain is not associated with the treprostinil dose (12). A long-term study that included 860 patients treated with SC treprostinil for PAH and followed for up to 4 years found that 92% of patients experienced infusion site pain at some point (the most common adverse event) and approximately 23% discontinued the study due to site pain (13). Current management of site pain has improved due to the pivotal study and anecdotal strategies for management of infusion site pain have been previously published and include strategies around site selection/rotation, dry insertion, and a variety of pre-medications, creams, gels (e.g., premium lecithin organogel (PLO) gel compounds), or patches (e.g., lidocaine and capsaicin) (11, 14, 15). Novel approaches may further help patients manage site pain and avoid treatment discontinuation. One such novel approach that is reported here is the use of cannabidiol (CBD) salve, a compound extracted from the hemp plant.

## Cannabidiol for pain

Cannabidiol is one of several compounds known as phytocannabinoids, where cannabinoids are derived from the female *Cannabis sativa* plant (16). Unlike another phytocannabinoid that has known psychoactive activity, ∆-9-tetrahydrocannabinol (THC), CBD is non-psychoactive and does not affect motor and cognitive functions or body temperature (16). The 2018 Farm Bill (United States) removed hemp as a Scheduled I controlled substance and led to the expansion of CBD-marketed products, with regulations on CBD varying from state to state (17, 18). As a result, hemp and its derivatives (including hemp-derived CBD) are no longer subject to regulation and oversight as a controlled substance by the United States Drug Enforcement Administration. The Farm Bill also expanded the statutory definition of what constitutes hemp to include, “all derivatives, extracts, cannabinoids, isomers, acids, salts, and salts of isomers,” if it contains no more than a 0.3% concentration of THC. Regarding pharmacologic therapeutics, the Food and Drug Administration (FDA) only requires premarket approval if the product claims an intended use of curing, mitigating, treating, or preventing a disease (18).

**Figure 1:**
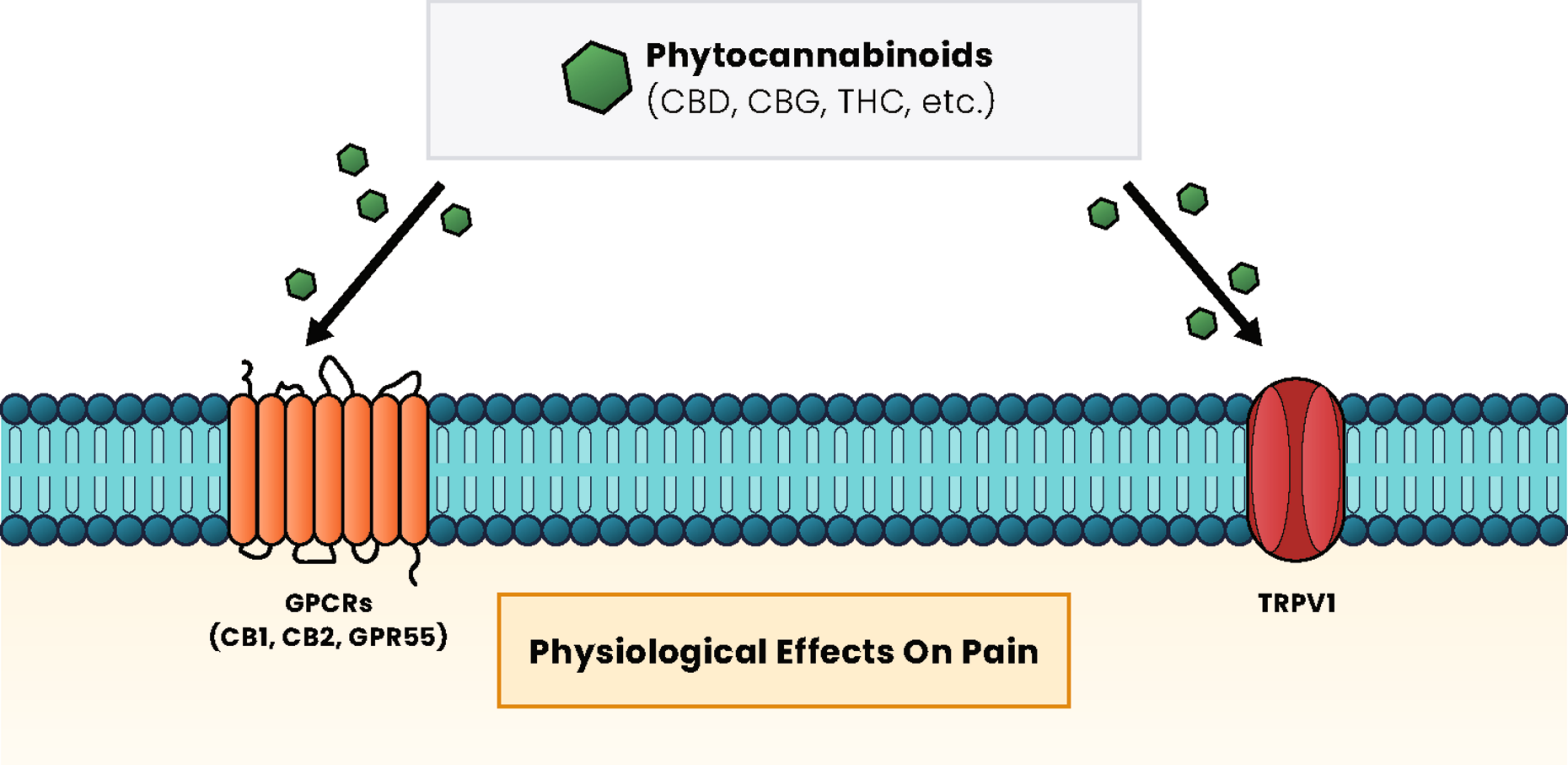
Cannabinoid signaling pathways and effects. Regardless of the type of cannabinoid ligand (phytocannabinoid, endocannabinoid, or synthetic), these compounds primarily interact with GPCR proteins, such as the CB1 and CB2 receptors and GPR55, or with TRP, such as TRPV1, to induce a cellular response. The pathways activated vary based on receptor activation and have physiological effects on pain, appetite, mood, and many other effects within the body. CB1, cannabinoid receptor type 1; CB2, cannabinoid receptor type 2; CBD, cannabidiol; CBG, cannabigerol; GPCRs, G protein-coupled receptors; MAPK, mitogen-activated protein kinases; PPAR, peroxisome proliferator-activated receptors; PI3K, phosphatidylinositol-3 kinase; THC, ∆-9-tetrahydrocannabinol.

The effects of phytocannabinoids are mediated through the endocannabinoid system (ECS) that is distributed throughout the body (16, 19). The ECS is a complex molecular and biological system that uses several compounds to regulate signaling pathways in areas like the brain, skin, blood vessels, immune cells, lungs, and liver. When an imbalance has been caused by pain and inflammation, cannabinoids have been suggested to help restore homeostasis through the ECS (19). CBD is a primary cannabinoid that can exert its effects within or outside of the ECS via many types of receptors (Figure 1) (20). Two ECS receptors are subtypes of G protein-coupled receptors (GPCRs) called cannabinoid receptor type 1 (CB1), which is mainly present in the nervous and immunological systems, and cannabinoid receptor type 2 (CB2), which is integral to cytokine release of immune cells (19, 20). CBD also regulates through multiple subfamilies of the transient receptor potential (TRP) cation channels (19). Preclinical studies suggest that TRPV1 receptors are upregulated in chronic pain models, and CBD administration can modulate the expression of this receptor (21, 22).

Cannabidiol is postulated to have analgesic, anti-inflammatory, and anxiolytic activities; however, there is limited scientific evidence to support these claims (23). Nonetheless, because of these effects, CBD is an attractive option for pain management while being non-euphoric and thus a low potential for abuse (18, 23). The anti-inflammatory aspect of CBD may be related to an analgesic effect as shown in controlled clinical studies (19, 24, 25). There are survey data that anecdotally suggest that CBD oil may alleviate SC site pain in pediatric PAH populations (15). However, the published literature on the use of CBD preparations for pain is mixed (26–28). Topical CBD in the form of lotions, salves, gels, and patches is one of several routes CBD may be delivered (29). Transdermal administration efficiently delivers medication directly to the local area where it is applied, thereby possibly reducing the dosage needed, decreasing side effects, and removing the need for systemic treatment (19).

## Case reports administering CBD for subcutaneous treprostinil site pain

In this case report, we detail the use of topical CBD for SC treprostinil site pain in two patient types. Both patients tried multiple palliative options offered by their care team and, after discussion and education, were interested in using topical CBD to address their pain while remaining on SC treprostinil.

### CBD case report #1

**Figure 2:**
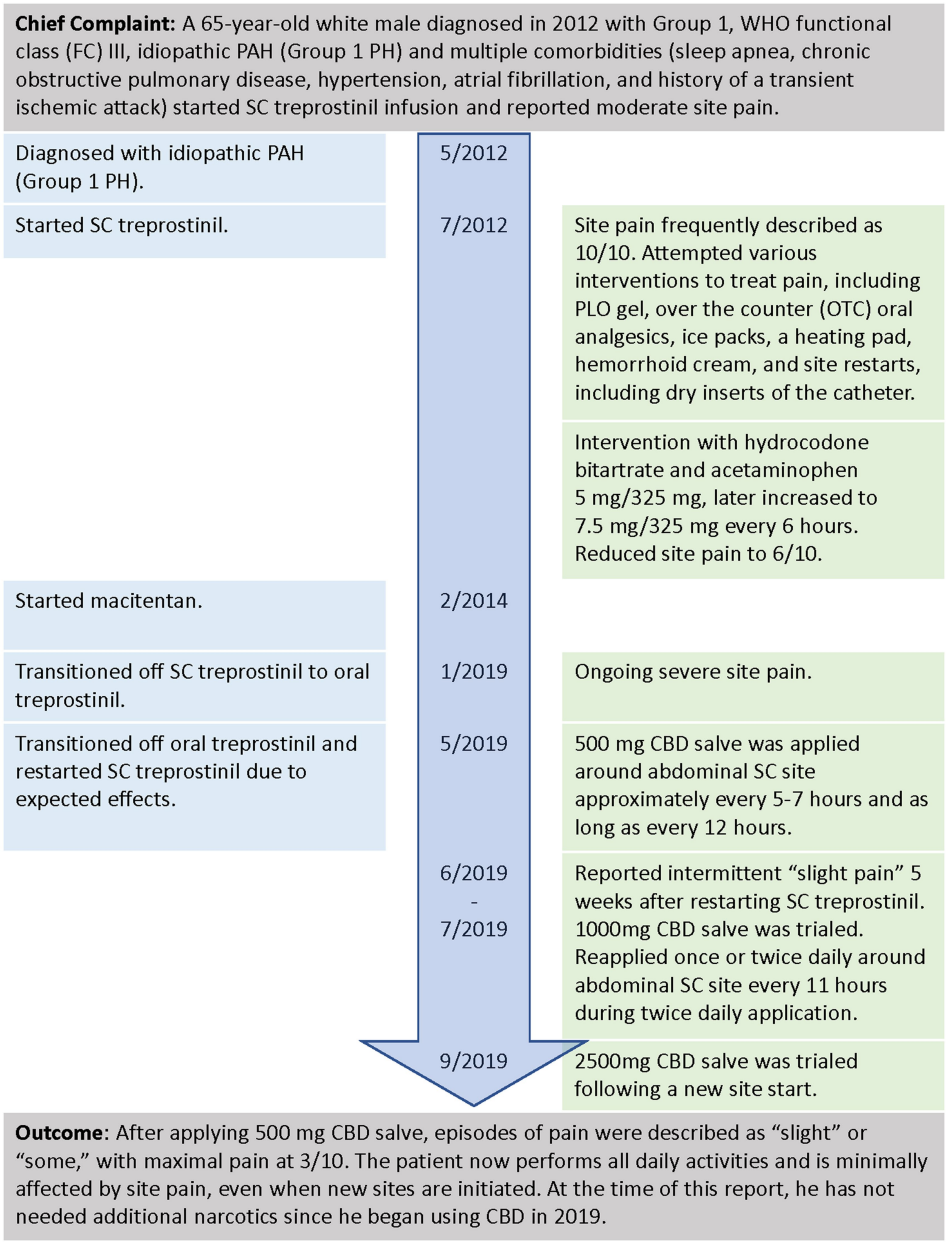
Timeline of interventions and outcomes for patient #1.

A 65-year-old white male diagnosed in 2012 with WHO functional class (FC) III, idiopathic PAH, and multiple comorbidities (sleep apnea, chronic obstructive pulmonary disease, hypertension, atrial fibrillation, and a history of a transient ischemic attack) started SC treprostinil infusion (Figure 2). He reported moderate site pain and attempted various interventions with the oversight of a nurse practitioner (NP). The intervention included a transdermal PLO gel compound (ketoprofen 10%, lidocaine 5%, gabapentin 6%, ketamine 5%, amitriptyline 2%, and clonidine 0.2%), over-the-counter (OTC) oral analgesics, ice packs, a heating pad, hemorrhoid cream, and site restarts, including dry inserts of the catheter. These interventions were insufficient as they offered only temporary relief, and the pain often continued to progress. At times, the site pain was unbearable, even interfering with his ability to complete daily activities. The decision was made to try oral narcotics, and the patient was initiated on hydrocodone bitartrate and acetaminophen 5 mg/325 mg, which was later increased to 7.5 mg/325 mg. While treprostinil infusion was increased to meet the initial goal of 40 ng/kg/min, the patient still complained of significant pain that affected his quality of life, as he was unable to walk due to excruciating pain.

After 7.5 years on SC treprostinil, despite oral narcotics and other interventions, the patient continued to describe intolerable acute site pain and decided to transition to oral treprostinil to treat his PAH. When the patient experienced side effects after taking oral treprostinil for 4 months, he contacted his PH team and asked to be transitioned back to SC treprostinil. The patient and PH team discussed transitioning back to SC treprostinil and agreed upon a plan for pain control that included the use of alternative and complementary measures prior to restarting any narcotics. The NP discussed topical CBD with the patient, reviewing its chemical compound, the possible need for the dose titration, and product availability.

Upon transition to SC treprostinil, the patient reported slight pain and redness at the site and started using 500 mg CBD salve topically near the site. The following day he did not notice any pain or redness. Then, as a prophylactic measure to prevent pain, he applied the CBD on any new site areas prior to placing the catheter, which he felt was successful at reducing pain levels to “mild to no pain.” When the pain was noted sporadically in the weeks after a new site was placed, CBD was applied 1–2 times in various doses and intervals to relieve site pain accordingly without the need for other pain-relieving methods.

Prior to using CBD, the patient described catheter site pain as “excruciating” or “intolerable” and a 10 out of 10 on the pain scale, with 10 being intense pain (30). Using oral narcotics reduced the pain to a 6 but with occasions of intolerable pain. With the CBD salve, episodes of pain were described as “slight” or “some,” with maximal pain at 3. The patient now performs all daily activities and is minimally affected by site pain, even when new sites are initiated. At the time of this report, he did not need additional narcotics since he began using CBD in 2019.

### CBD case report #2

**Figure 3:**
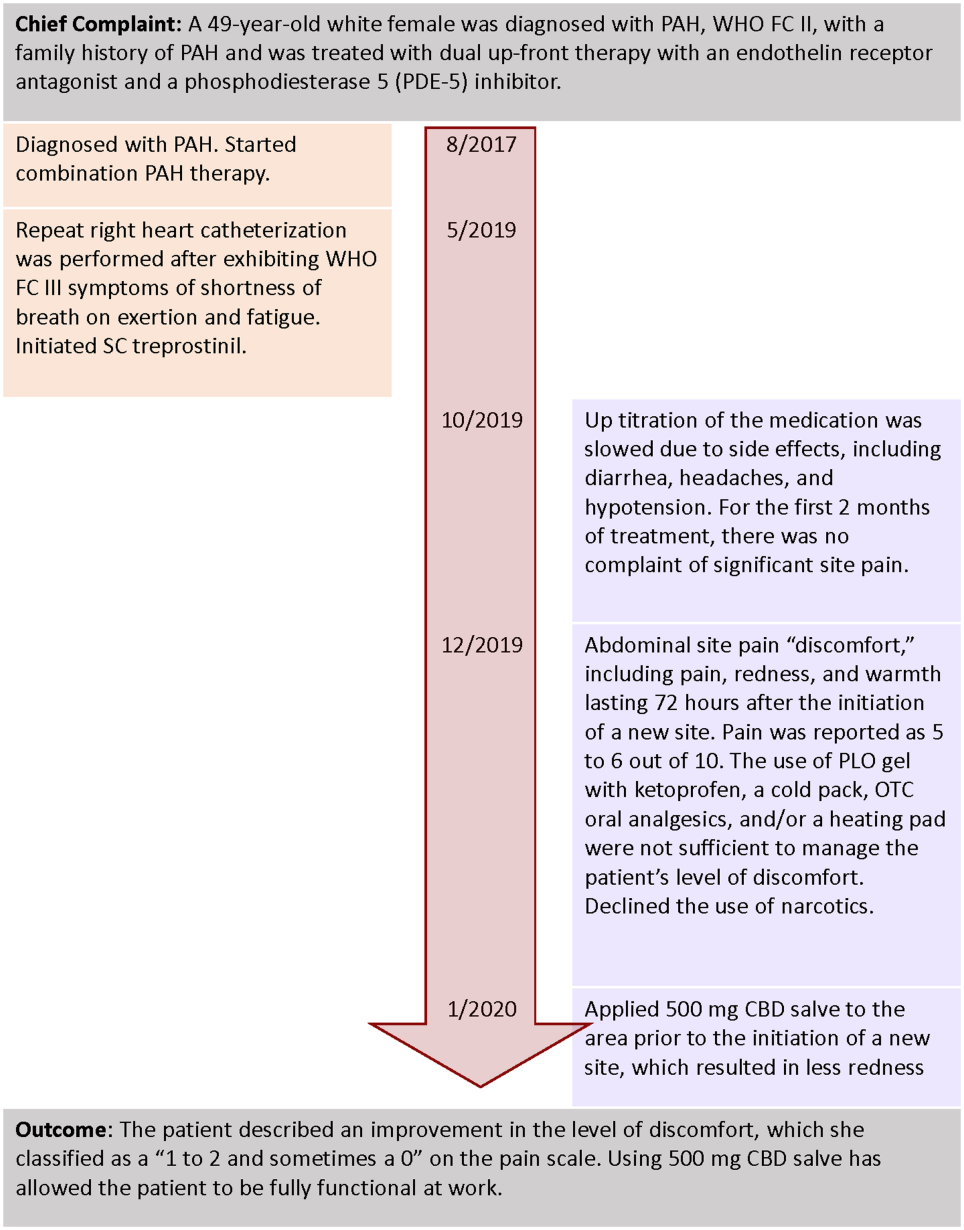
Timeline of interventions and outcomes for patient #2.

A 49-year-old white female was diagnosed with PAH, WHO FC II, with a family history of PAH (Figure 3). Her comorbidities included sleep apnea, morbid obesity, depression, and anxiety with a history of alcohol use disorder and smoking. She was treated with dual upfront therapy with an endothelin receptor antagonist and a phosphodiesterase 5 (PDE-5) inhibitor. After 2 years of receiving combination treatment, she started to exhibit WHO FC III symptoms of shortness of breath on exertion and fatigue. A repeat right heart catheterization was performed, and parenteral prostacyclin therapy for worsening PAH was initiated.

Upon discussion with the patient, the treatment team decided to initiate SC treprostinil. The initial infusion site was initiated in the anterior abdominal wall using dry catheter insertion. Up titration of the medication was slowed due to side effects, including diarrhea, headaches, and hypotension. The patient used the same SC site for 4–6 weeks before starting a new site. For the first 2 months of treatment, there were no complaints of significant site pain. After 2 months, the patient described abdominal site pain “discomfort,” including acute pain, redness, and warmth lasting 72 h after the initiation of a new site. On a scale of 0–10 with 10 being intense pain, the patient rated the site pain as a 5–6 (30). The use of PLO gel with ketoprofen, a cold pack, OTC oral analgesics, and/or a heating pad was not sufficient to manage the patient’s level of discomfort.

The abdominal discomfort and pain affected the patient’s ability to sit for more than 1 h and ambulate after the initiation of a new site. The patient declined the use of oral narcotics.

Alternatives to manage pain without the use of prescription narcotics were discussed, with a focus on topical CBD salve. The patient and NP discussed CBD, the potential to titrate the dose, the cost of treatment, and questions surrounding its use. After a new SC site was started, the patient self-adjusted the dosing and timing of CBD applications around the site, based on her level of pain and redness. She also applied 500 mg CBD salve to the area prior to the initiation of a new site, which resulted in less redness. The patient described an improvement in the level of discomfort, which she classified as a “1–2 and sometimes a 0” on the pain scale (30). Using CBD salve has allowed the patient to be fully functional at work.

## Discussion

Subcutaneous treprostinil is effective for the treatment of PAH, but therapy may be limited by site pain and reaction, leading to discontinuation that ranges from 8%–23% of patients in clinical studies (12, 13). There are many strategies to mitigate site pain including appropriate site selection/rotation, dry insertion, and various pre-medications, creams, or patches; however, novel approaches are warranted (11, 14, 15). Here, we present data from two patients with moderate-to-severe site pain and reaction that were successfully managed with CBD preparations. Both patients discovered successful strategies to reduce infusion site pain, which included the application of a low-dose CBD salve to a new site, applying more CBD salve, or uptitrating the dose, as needed for persistent pain.

Teams treating a patient with PAH with SC treprostinil should set reasonable expectations and present pain management options. Given the concerns related to the addiction potential for narcotics, many providers may suggest a variety of options for pain management. In these cases, topical CBD could be successful with patients willing to try multiple strategies and doses to find the methods that work best for them. However, the conversation about CBD can occur at any time in SC treprostinil education, not only when other methods are exhausted. Topical application is the preferred route of administration for CBD to limit systemic exposure (31). These results suggest the potential for the use of CBD salve, one of the multiple forms of topical CBD, to alleviate SC site pain.

Studies and published data are not available for describing the mechanism by which CBD manages pain in the PAH population, thus no recommendations are available. Similarly, this is also the case for other anecdotal methods of SC site pain management currently used. Access to high-quality CBD may be a challenge for patients, as well as its cost since, to date, it is not likely to be covered by insurance and is not covered by Medicare. Additional education about CBD products and their lack of psychoactive effects is needed to help both patients and clinicians understand the safety and potential efficacy of CBD products (27).

Although the mechanisms for CBD and pain management in the PAH population have not been studied in detail, there is some literature generally relating to CBD and pain. CBD is known to interact with a variety of molecular targets including enzymes and ion channels/ionotropic receptors (25). In animal models, CBD has analgesic effects through varied administration routes (25, 32). CBD gel and ointment studies have seen positive results (19, 32). In a rat model of arthritis, transdermal CBD gel significantly reduced pain behaviors, joint swelling, immune cell infiltration, and thickening of the synovial membrane in a dose-dependent manner (32). A spontaneous, anecdotal, retrospective study of 20 human patients with inflammatory diseases, such as psoriasis and dermatitis, administered CBD ointment and found improved skin hydration and elasticity, as well as improved quality of life (33).

As with any medication, there is the potential for CBD to interact with SC treprostinil. However, no research studies have looked at this potential interaction in this population. Using a transdermal application directly to the location of the site, the potential for systemic effects of CBD is lessened (31). In these patients, the choice of CBD salve was made to treat site pain, although as mentioned above, there are other topical options for treating site pain such as high-dose (8%) capsaicin, a TRPV1 agonist, administered as a patch (34, 35). Capsaicin and CBD may lessen pain through shared pathways or mechanisms as they both modulate the TRPV1 receptor, which is known to play a role in pain (21, 22).

## Limitations

This study only presents two case reports from a single center. Detailed CBD salve composition, including medication strength, was not listed by the manufacturer and may affect treatment outcomes. Structured randomized control clinical trials are needed to sufficiently evaluate whether CBD can be an efficacious treatment for SC treprostinil infusion site pain.

## Conclusion

Successful use of CBD to minimize redness and alleviate pain in the following case studies suggests that topical CBD may be an effective, non-psychoactive, non-addictive treatment for SC treprostinil infusion site pain. Transdermal administration of CBD allows its application directly at the SC infusion site and also allows for dose adjustments according to the treatment needs of the patient. The two case reports presented here found that both patients were able to resume their daily lives with the use of topical CBD to reduce SC infusion site pain and redness, but further testing of CBD to address this common challenge is needed within the PAH community.

## Data availability statement

The original contributions presented in the study are included in the article/supplementary material, further inquiries can be directed to the corresponding author.

## Ethics statement

Written informed consent was obtained from the individual(s) for the publication of any potentially identifiable images or data included in this article.

## Author contributions

AK and JB contributed to the drafting of the manuscript. JB completed patient examinations, recommended treatment options, and oversaw case management. All authors read, edited, and approved the final version of the manuscript.

## Funding

This medical writing support was provided by United Therapeutics.

## Conflict of interest

AK is an employee of United Therapeutics Corporation. JB is on the speaker’s bureau for United Therapeutics Corporation and Janssen.

## Publisher’s note

All claims expressed in this article are solely those of the authors and do not necessarily represent those of their affiliated organizations, or those of the publisher, the editors and the reviewers. Any product that may be evaluated in this article, or claim that may be made by its manufacturer, is not guaranteed or endorsed by the publisher.
